# The Elusive Diagnosis of Primary Esophageal Lymphoma

**DOI:** 10.4274/tjh.2016.0482

**Published:** 2018-08-05

**Authors:** Rachel Abou Mrad, Nadim El-Majzoub, Ali Shamseddine, Assaad Soweid

**Affiliations:** 1 American University of Beirut Medical Center, Department of Internal Medicine, Division of Gastroenterology and Hepatology, Beirut, Lebanon; 2 American University of Beirut Medical Center, Department of Pathology and Laboratory Medicine, Beirut, Lebanon; 3 American University of Beirut Medical Center, Department of Internal Medicine, the Division of Hematology and Oncology, Beirut, Lebanon

**Keywords:** Endoscopy, Endosonography, Esophagus, Non-Hodgkin lymphoma

A 76-year-old woman presented with a 2-month history of progressive dysphagia that was associated with weight loss. Computed tomography of the neck showed significant circumferential soft tissue thickening involving the upper esophagus with luminal narrowing (Figure 1A). Upper gastrointestinal endoscopy revealed a very tight stricture below the cricopharyngeus muscle. The stricture was traversed using a neonatal endoscope. Endoscopic ultrasonography using a miniprobe revealed marked esophageal wall thickening with diffuse hypoechoic infiltration involving the entire wall (Figure 1B). Biopsy specimens from the esophageal stricture revealed malignant non-Hodgkin lymphoma (diffuse large B-cell type) confirmed by immunohistochemistry Figure 1C: hematoxylin and eosin staining at 100^x^ magnification of the lymphoid infiltration; Figure 1D: Ki67 (proliferation index) staining at 400^x^, 40% tumor cells). The patient received 6 cycles of chemotherapy [anti-CD20 monoclonal antibody (rituximab) plus the CVP regimen], followed by positron emission tomography/computed tomography and upper endoscopy with a biopsy that showed no evidence of lymphoma.

## Figures and Tables

**Figure 1:**
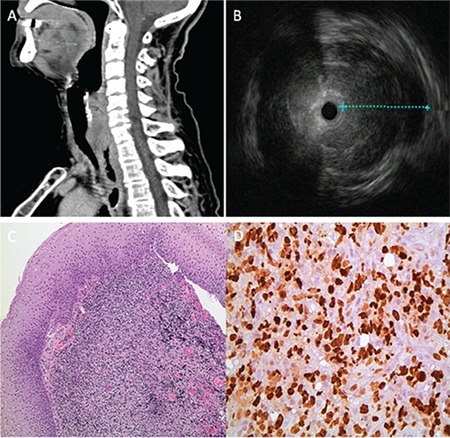
Diagnosis of primary esophageal lymphoma.

